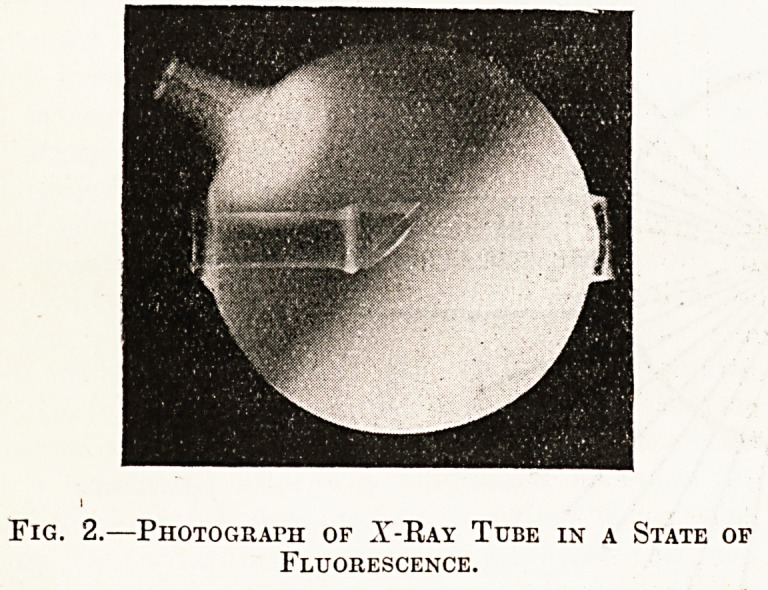# The X-Rays

**Published:** 1912-06-08

**Authors:** Alfred C. Norman

**Affiliations:** House Surgeon at Sunderland and Durham County Eye Infirmary, Sunderland.


### ELECTRICITY IN MODERN MEDICINE.*

### XIII.—The X-Rays.

By ALFRED C. NORMAN, M.D. Edin., House Surgeon at Sunderland and Durham County Eye Infirmary, Sunderland.

### Properties of the Cathode Rays.

The cathode rays may be regarded as a stream of minute material particles (*electrons*) thrown off from the cathode at right angles to its surface and travelling away from it in straight lines at a velocity of about 19,000 miles per second—*i.e.* about one-tenth the velocity of light. The particles are quite invisible, of course (their mass being but $\frac{1}{1700}$ of that of a hydrogen atom), they carry a negative charge, and where they strike the walls of the tube they produce fluorescence and a considerable amount of heat; in fact, if they are brought to a focus they will quickly melt a hole in the glass or even in a metal disc. Of greater importance to us, however, is the fact that when they strike a solid body, such as the walls of the tube or a platinum disc within the tube, *x*-rays are generated. The higher the vacuum of the tube the greater is the velocity of the cathode stream and the more penetrating are the *x*-rays. The cathode rays do not penetrate the walls of an ordinary Crookes tube, they do all their work inside; but the *x*-rays are sufficiently penetrating to pass out of the tube in the case of all tubes of the Crookes degree of vacuum.

In the primitive Crookes tube with wire electrodes described in the last section it is obvious that *x*-rays are generated in an irregular fashion in many parts of the tube and that their direction is very little under the control of the operator. It was found, however, that by shaping the cathode like a concave mirror the cathode rays could be brought to a focus in the centre of the tube, and that a flat disc of platinum placed in the centre of the tube would give out *x*-rays in a regular manner from the single point on its surface to which the cathode rays were focussed. Unless the *x*-rays emerge from a single point it is impossible for them to produce a sharp radiogram (just as the light from many candles would not produce a clear shadow); it was the introduction of the "focus tube," as it is called, that advanced radiography to the position of an exact science.

### The Modern X-Ray Focus Tube.

Fig. 1 illustrates diagrammatically the parts of a modern focus tube. It consists of a circular glass bulb (varying in different tubes from $4\frac{1}{2}$ to 8 inches in diameter), with three hollow prolongations or stems into which the electrodes are fused, the whole tube being exhausted to less than $\frac{1}{1000000}$ atmosphere. The cathode is fused into the shorter of the main stems and terminates in an aluminium disc shaped like a concave mirror; it does not project into the bulbous part of the tube at all. The target or anticathode (so called because it is situated opposite the cathode and bears the brunt of its discharge) consists of a flat disc of platinum; it is always fixed in the centre of the tube and is inclined at an angle of 45° to the long axis of the tube. The anticathode in most tubes also functions as the anode and is simply the termination of that electrode. In the type of tube shown in the diagram the metal rod which supports the anticathode is fused into the long stem of the tube and is surrounded by a funnel of sheet iron to radiate away the heat generated by the impact of the cathode rays. In many tubes there is an accessory anode (marked "Anode" in the diagram) fused into a third stem of the tube; it is only of use when the tube is being exhausted by the makers. In some tubes the accessory anode is simply a straight metal rod, in others it is placed behind instead of above the anticathode; in fact this

Fig. 1.—Diagram of X-Ray Tube showing Path of Cathode Stream and Reflection of X-Rays.

[*By kind permission of Mr. Schall.*]

* Previous articles appeared on Nov. 11, 25, Dec. 9, 30, Jan. 13, 27, Feb. 17, March 9, 30, April 20, May 4, 25.

electrode is of very little importance once the tube has left the makers' hands.

It is of the utmost importance, however, that the user connect the tube properly to the induction coil, otherwise the former will be spoilt in a very short time. He should remember that the anticathode has two functions—it is the active *anode* of the tube, as well as being the *target* upon which the cathode stream impinges—and he should always connect it with the positive pillar (anode) of the induction coil. The cathode must, of course, be connected with the negative pillar of the coil (as a useful mnemonic, it may be noted that the "*concave* electrode of the tube must be *connected* with the *cathodal* pillar of the *coil*").

When the tube is properly connected with the induction coil, according to our present ideas, positive electricity enters at the anode and leaves at the cathode, and in doing so gives rise to other phenomena which are diagrammatically illustrated in fig. 1. The cathode stream of negatively charged particles (indicated by broken lines in the figure) is discharged at a tremendous velocity from the concave

Fig. 2.—Photograph of X-Ray Tube in a State of Fluorescence.

surface of the cathode, and is brought to a focus at some point near the centre of the obliquely inclined anticathode. At this point $x$-rays are generated and discharged in all directions in front of the anticathode (as indicated by the continuous lines in the figure). The $x$-rays are of course invisible, but where they strike the walls of the tube they excite a beautiful greenish fluorescence, and since they are only able to reach that part of the tube which lies in front of the plane of the anticathode the result is that the portion of the tube in front of this plane is brightly illuminated, whilst the part behind is nearly dark. When a tube is thus sharply differentiated into a light and a dark portion the user knows that it has been properly connected to the coil, that it is working to the best advantage, and that there is practically no reverse current passing through it. On the other hand, when a tube is connected to the coil the wrong way round (*i.e.* the cathode of the tube to the positive pillar of the induction coil) the anticathode has to play the part of cathode, and, in consequence, the cathode rays emitted from its flat surface are not brought to a focus, but are scattered to various parts of the tube, where they excite an irregular fluorescence in the glass walls, and the tube is seen to be covered by irregular patches and streaks of light having no definite arrangement. This is, of course, a condition of complete reverse current. A condition similar, but not so well marked, is produced in a tube (properly connected to the coil) if the latter be generating a considerable amount of reverse current. In this case the anticathode is only acting as cathode in so far as the reverse current is concerned, but this is quite sufficient to produce a certain amount of irregular fluorescence in the glass walls of the tube, and as a consequence the sharp line of demarcation between the light and dark portions is more or less obscured.

Fig. 2 shows the typical appearance of an $x$-ray tube working satisfactorily, as seen in the dark room. All $x$-rays were carefully screened from the camera so that the illustration is really nothing more than a photograph of a tube in a state of fluorescence. A type of tube similar to the diagram in fig. 1 (except that the accessory anode is represented by a rod of aluminium) was chosen for the purpose. In the photograph the outline of the bulbous portion of the tube can be made out, but the two main stems are invisible, except just at their junction with the tube, because they are not fluorescing. The anticathode, projecting into the bulbous portion of the tube, can be seen and the concave cathode is also just visible. The important point to notice, however, is that the part of the tube in front of the plane of the anticathode is brilliantly illuminated whilst the part behind that plane is dark, and that there is a fairly well-defined line of demarcation between the two areas. This indicates that the tube is correctly connected to the coil and that there is very little reverse current. That there is some reverse current, however, is shown by the fact that the small stem of the tube (above the anticathode stem) is in a state of fluorescence. This stem contains the rod-shaped accessory anode, and the latter, by virtue of the reverse current, is emitting some cathode rays, which are causing the stem and the part of the bulb in its vicinity to fluoresce.

The anticathode of a modern tube is always set at an angle of 45° to the long axis of the tube in order to discharge $x$-rays through one *side* of the tube instead of through the *end*. (If the anticathode were set squarely opposite the cathode the central $x$-rays would be discharged through the cathode end of the tube, and the cathode would throw a large shadow on the photographic plate or fluorescent screen.) In arranging a tube to take a radiogram it is only necessary to remember that $x$-rays are being discharged in straight lines from that half of the tube which is fluorescing brightly and uniformly, and that this portion of the tube should be directed towards the patient in such a way that *the long axis of the tube is parallel with the surface of the photographic plate*. There is nothing to be gained by tilting the tube, as is sometimes done, so that the surface of the anticathode is parallel with the plate.

(*To be continued.*)

## Figures and Tables

**Fig. 1. f1:**
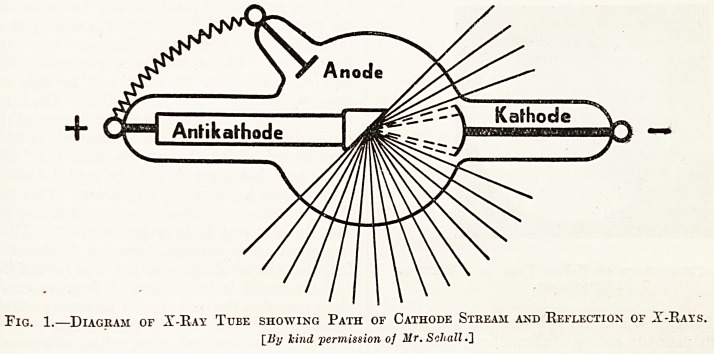


**Fig. 2. f2:**